# Mesoporous Silica-Based Nanoparticles as Non-Viral Gene Delivery Platform for Treating Retinitis Pigmentosa

**DOI:** 10.3390/jcm11082170

**Published:** 2022-04-13

**Authors:** Lourdes Valdés-Sánchez, Sara Borrego-González, Adoración Montero-Sánchez, Simone Massalini, Berta de la Cerda, Aránzazu Díaz-Cuenca, Francisco J. Díaz-Corrales

**Affiliations:** 1 Regeneration and Cell Therapy Department, Andalusian Molecular Biology and Regenerative Medicine Centre (CABIMER), Junta de Andalucía, CSIC, Universidad de Sevilla, Universidad Pablo de Olavide, 41092 Sevilla, Spain; lourdes.valdes@cabimer.es (L.V.-S.); dori.montero@cabimer.es (A.M.-S.); s.massalini@prinsesmaximacentrum.nl (S.M.); 2 Materials Science Institute of Seville (ICMS), Joint CSIC-University of Seville Center, 41092 Seville, Spain; sarbogo@gmail.com; 3 Networking Research Center on Bioengineering, Biomaterials, and Nanomedicine (CIBER-BBN), 28029 Madrid, Spain

**Keywords:** retinitis pigmentosa, *PRPF31*, gene therapy, nanoparticles, mesoporous silica

## Abstract

Background: Gene therapy is a therapeutic possibility for retinitis pigmentosa (RP), in which therapeutic transgenes are currently delivered to the retina by adeno-associated viral vectors (AAVs). Although their safety and efficacy have been demonstrated in both clinical and preclinical settings, AAVs present some technical handicaps, such as limited cargo capacity and possible immunogenicity in repetitive doses. The development of alternative, non-viral delivery platforms like nanoparticles is of great interest to extend the application of gene therapy for RP. Methods: Amino-functionalized mesoporous silica-based nanoparticles (N-MSiNPs) were synthesized, physico-chemically characterized, and evaluated as gene delivery systems for human cells in vitro and for retinal cells in vivo. Transgene expression was evaluated by WB and immunofluorescence. The safety evaluation of mice subjected to subretinal injection was assessed by ophthalmological tests (electroretinogram, funduscopy, tomography, and optokinetic test). Results: N-MSiNPs delivered transgenes to human cells in vitro and to retinal cells in vivo. No adverse effects were detected for the integrity of the retinal tissue or the visual function of treated eyes. N-MSiNPs were able to deliver a therapeutic transgene candidate for RP, *PRPF31*, both in vitro and in vivo. Conclusions: N-MSiNPs are safe for retinal delivery and thus a potential alternative to viral vectors.

## 1. Introduction

Retinitis pigmentosa (RP) is the leading cause of inherited blindness in adults, affecting 1:4000 people worldwide [1]. RP begins with progressive rod photoreceptor degeneration, which initially manifests as night blindness. As the degenerative process advances, the function of the peripheral retina declines, causing loss of the peripheral visual field and tunnel vision. In the disease’s advanced stages, loss of central vision and absolute blindness occurs. So far, mutations in more than 80 genes have been implicated in non-syndromic RP [1]. Many of these genes encode retinal-specific proteins such as rhodopsin, but others are ubiquitously expressed, such as the pre-mRNA splicing factors PRPF3, PRPF4, PRPF6, PRPF8, and PRPF31 [2,3]. Mutations in the *PRPF31* gene are a common cause of autosomal dominant retinitis pigmentosa [4]. Curiously, in mutant Prpf31 mouse models, the cell layer of the retina that is primarily affected is the retinal pigment epithelium (RPE) [5]. The molecular mechanisms by which mutations in the ubiquitously expressed *PRPF31* gene induce retinal degeneration remain unclear and controversial. Haploinsufficiency alone or combined with the dominant-negative effect of the mutant protein in the RPE cells has been proposed as the possible pathophysiological mechanism [6,7,8]. Therefore, a standard gene augmentation therapy may not be sufficient to treat this form of RP, and the delivery of both a normal copy of the *PRPF31* gene and a silencer for the mutated allele might be necessary.

Gene therapy is a very promising emerging treatment for degenerative pathologies of the retina. In patients affected by Leber’s congenital amaurosis and RP caused by mutations in the *RPE65* gene, successful gene therapy clinical trials have been completed, in which a viral vector containing the *RPE65* transgene was injected into the subretinal space [9,10]. Since 2017, with the approval of *RPE65* gene therapy in the USA (STN:125610) and Europe (EMA/831436/2018), the interest in gene therapy for retinal dystrophies has been renewed. Adenoviral, lentiviral, and adeno-associated viral (AAV) vectors have been used to transduce ocular tissues efficiently, with AAV vectors the most widely used due to their serotypes distinctively targeting different cells of the neural system and their low antigenicity and low risk of integration in the host genome [11]. However, AAVs are challenging to handle, costly to produce and scale-up, and it is under debate if the presence of neutralizing antibodies in treated patients would decrease the transfection efficiency in subsequent treatments [12,13]. In addition, their limited DNA packing capacity prevents the delivery of DNA over 5 kb. Dual gene therapy requiring delivery of a coding sequence and silencer is an additional difficulty for AAVs. For this reason, the development of new non-viral vectors such as nanoparticles might constitute a valuable alternative to AAVs.

Lipidic, polymeric, or metallic nanoparticles have been proposed as gene delivery platforms for retinal therapies [14]. Nanoparticles with the lipidic core of Precirol ATO5 carrying the RS1 gene led to restoring the retinal structure in a retinoschisis-deficient mouse [15]. Other studies have tested polymeric nanoparticles, such as CK30-PEG polymer-based nanoparticles [16] or the dendrimers of modified hydroxy-terminated polyamidoamine as fabricated by Tai et al. [17], which improved the permeability of oligonucleotides and prolonged their distribution in the retina for non-invasive intraocular delivery. Non-cytotoxic metallic gold nanoparticles have also shown the capacity to deliver plasmid DNA into RPE cells [18].

Other interesting options are mesoporous silica-based nanomaterials, which can offer good biocompatibility [19,20,21,22,23,24] and can be engineered in the form of nanoparticles for gene delivery [25,26,27]. Silica has been “generally recognized as safe” according to the U.S. Food and Drug Administration (FDA) for over 50 years, and silica-based nanomaterials are thus especially attractive for their high biocompatibility, nontoxic degradation products, and tuneable hydrolytic degradability in a biologically relevant environment from several hours to days or weeks [23]. Mesoporous silica nanoparticles are unstable in water media and dissolve into silicic acid molecules (Si(OH)_4_), which are soluble in aqueous media and excreted in the urine, providing a material with excellent bioavailability without toxicity [23]. Although the translation to the clinic remains challenging, mesoporous silica-based nanoparticles (MSiNPs) are recognized as one of the most promising nanocarriers and are being considered in a wide range of medical applications [28,29]. MSiNPs’ preeminent attributes are the high surface area (400–1000 m^2^ g^−1^), nanopore volume (0.6–1.0 cm^3^ g^−1^), and modifiable pore size (2–20 nm), which make them powerful therapeutic vehicles for the drug delivery of antibiotics, growth factors, and nucleic acids [30,31]. In addition, their textural properties can be adjusted and controlled by varying the synthesis parameters [26], and the silica (SiO_2_) chemistry allows not only the incorporation of Ca, P, Fe, Zr, Mn, and other elements to obtain different properties [23] but also a simple surface functionalization [32].

Although the application of MSiNPs in the treatment of ocular diseases such as retinoblastoma [33,34,35,36] is starting to be reported [37], the use of MSiNPs in the context of non-viral delivery for gene therapy in retinal diseases is still unexplored. MSiNPs are typically modified with surface amination to improve the binding to negatively charged nucleic acids. Therefore, the goal of the present study was to evaluate the possible gene-therapy application of the aminofunctioalized MSiNPs (N-MSiNPs) for the treatment of retinal dystrophies, specifically RP. For this purpose, we analyzed the transfection capacity of these nanosystems and their functional effects in vitro, in human cell culture, and in vivo after subretinal injection in mice.

## 2. Materials and Methods

### 2.1. Preparation of the Mesoporous Silica-Based Nanoparticles (MSiNPs) and Amino-Functionalization

Mesoporous silica-based nanoparticles (MSiNPs) were synthesized by the sol-gel method using ethyl acetate (EA)-hexadecyltrimethyl ammonium bromide (CTAB)-water microemulsion droplets as a soft template, according to the protocol reported by Liang et al. [38]. Firstly, 0.7 g of hexadecyltrimethyl ammonium bromide (CTAB, A15235, Alfa-Aesar, Karlsruhe, Germany) was dissolved in 33 mL of dH_2_O, using an oil-bath at 30 °C and 800 r.p.m. When CTAB dissolved completely (15 min), 10 mL of ethyl acetate (EA, 270989, Sigma-Aldrich, Saint Louis, MO, USA) was added. After 30 min stirring, 7 mL (1 mol L^−1^) aqueous ammonia (17093, Fluka & Sigma-Aldrich Chemie; Steinheim, Germany) was added. Then, the mixture was stirred for 15 min, and 3.6 mL tetraethyl orthosilicate (TEOS, 14082, Alfa-Aesar), 0.36 mL triethyl phosphate (TEP, 538728, Sigma-Aldrich), and 2.277 g calcium nitrate tetrahydrate (CN, C4955, Sigma-Aldrich) were sequentially added to the above mixture at 30 min intervals. The resulting solution was then vigorously stirred for 4 h. The white precipitate was collected by centrifugation and washed three times with ethanol and dH_2_O in turn. Then, the precipitate was frozen at −20 °C and lyophilized for 48 h. Finally, the samples were obtained after removing organics and nitrates by calcination at 650 °C using a ramp of 2 °C min^−1^ to obtain the final MSiNPs product. The MSiNPs were amino-functionalized (−NH_2_ groups) using 3-aminopropyl triethoxysilane (APTES) [39]. First, 0.1 g of MSiNP powder was dispersed in 50 mL of toluene (1.08323, Emplura, Merck, Darmstadt, Germany), and then 1 mL of APTES (A3648, Sigma-Aldrich) was added and refluxed at 80 °C for 24 h to obtain the N-MSiNP final product. The N-MSiNPs were collected by centrifugation at 10,000 r.p.m. for 5 min and washed with toluene and ethanol. Finally, the N-MSiNPs were dried in an oven at 80 °C for 24 h.

### 2.2. Nanoparticle Physicochemical Characterization

The compositional analysis of the MSiNPs was measured by X-ray fluorescence (XRF) using the Spectrometer Panalytical AXIOS model. Transmission electron microscopy (TEM) observations were performed using a JEOL 2100 Plus working at an accelerating voltage of 200 kV. The particle size distribution was measured from TEM-recorded images using ImageJ software. The textural parameters and the nanoporosity were measured by N_2_ adsorption–desorption on a Micromeritics Tristar 3020 gas adsorption analyzer at 77 K after degassing the samples at 523 K (250 °C) for 2 h in a nitrogen stream. The textural parameters, surface area, and nanopore size were calculated according to the accepted methods in the literature [40]. The chemical bonds and functional groups, before and after amino functionalization, were evaluated by Fourier transform infrared (FT-IR) spectroscopy using a JASCO 6200 spectrometer in a transmission configuration. The amino functionalization of the nanoparticles was also qualitatively tested using salicylaldehyde (84160, Fluka & Sigma-Aldrich; Saint Louis, MO, USA) [41].

### 2.3. DNA Loading

The ability of nanoparticles to load DNA in the form of a plasmid containing all the elements necessary for the expression of an exogenous transgene in human cells was evaluated. The plasmid pEGFP-N1 (Clontech Laboratories, Inc., Kusatsu, Shiga, Japan) was used, which encodes a red-shifted variant of wild-type GFP with a total plasmid size of 4.7 kb. Once the N-MSiNPs were autoclaved, they were dispersed in 1% PBS using an ultrasonic bath for 15 min. Then, 1 µg of plasmid pEGFP-N1 was mixed with 300, 150, 75, 37.5, or 18 µg of nanoparticles in 200 µL of 1% PBS and incubated for 1 h with orbital shaking at room temperature to find the optimal DNA:N-MSiNP ratio and achieve the highest charging efficiency. For evaluation, the charged particles and the controls were subjected to gel electrophoresis. Agarose gels were prepared at a concentration of 1% (*m*/*v*) in Tris-borate-EDTA containing 0.05 µL/mL of RedSafe. Then, 100 V was applied for 30 min. Free pEGFP-N1 and non-functionalized MSiNPs were used as controls.

### 2.4. Cell Culture

Cell line HEK-293 was kept in culture at 37 °C in a humid chamber with 5% CO_2_ and grown in Dulbecco’s modified Eagle’s medium F12 (DMEM/F12; Sigma-Aldrich) supplemented with 1% penicillin/streptomycin (Sigma-Aldrich), 1% glutamine (Sigma-Aldrich), and 10% fetal bovine serum (Sigma-Aldrich). The culture medium was changed every 2 days. Transfection positive control was performed using Lipofectamine 2000 (Invitrogen, Walthan, MA, USA) as a standard transfection substrate, with a 3:1 (μL of Lipofectamine 2000/μg of DNA) ratio, according to the manufacturer’s instructions. Briefly, 7.5 × 10^5^ cells were seeded in a 6 cm culture dish (Orange Scientific, Braine-l´Alleud, Belgium), and 24 h after seeding, cells were transfected with 180 µg of N-MSiNP nanoparticles complexed with 1 µg of PRPF31-GFP plasmid. PRPF31-GFP plasmid was prepared [42] with the open reading frame of *PRPF31* gene fused in phase with the N-terminus of GFP in the expression vector pEGFP-N1. Twenty-four-hours post-transfection, cells were either fixed or collected for protein isolation, depending on the experiment.

### 2.5. Western Blot

Proteins were extracted in ice-cold RIPA buffer containing a protease inhibitor cocktail. Thirty micrograms of each extract were separated in a denaturing 4–20% SDS–PAGE gel (Biorad, Hercules, CA, USA), and the proteins were transferred to a PVDF membrane (Amersham Biosciences, Little Chalfont, UK) and blocked using Superblock Blocking buffer (Thermo Fisher Scientific, Waltham, MA, USA) containing 0.1% of Tween-20 (Sigma-Aldrich) for 1 h at room temperature. The primary antibodies, anti-PRF31 (1:3000, Santa Cruz Biotechnology, SC-68347), anti-GAPDH (1:1000, ab9484, Abcam, Cambridge, UK), and anti-GFP (1:500; 2956, Cell Signaling, Danvers, MA, USA), were incubated overnight at 4 °C. The membrane was probed with the appropriate anti-HRP-conjugated secondary antibodies for 1 h at room temperature, and the immunoreactive bands were detected by chemiluminescence using ECL plus (Amersham Biosciences, Amersham, UK).

### 2.6. Animal Handling

All experiments described in this work were performed in compliance with the Spanish and European Union laws on animal care in experimentation (EU Directive 2010/63/EU). The animal manipulation and experimental methods of our laboratory were analyzed and approved by the Committee of Animal Experimentation of CABIMER (Seville, Spain) and by the Andalusia Local Government (Junta de Andalucía, Spain). All efforts were made to minimize the number of animals used and their suffering. Mice were housed in the Biological Resources Unit of CABIMER and kept in a temperature-controlled environment (21 ± 1 °C) with a relative humidity of 55 ± 5% and a light/dark cycle of 08:00–20:00 and given standard mouse chow and water ad libitum.

### 2.7. Subretinal Injection

Surgical procedures were performed under general anesthesia by the intraperitoneal injection of ketamine hydrochloride and xylazine solution (100/10 mg/kg body weight). The eyes were topically anesthetized with 0.1% tetracaine and 0.4% oxybuprocaine. The pupils were dilated with one drop of each 10% phenylephrine and 1% tropicamide. A 32-gauge needle was used to gently open the choroid 1 mm posterior to the sclerocorneal limbus. One single injection with a 10 μL syringe (Hamilton, Switzerland) with a 33-gauge needle attached to an ultramicropump (World Precision Instruments, Sarasota, FL, USA) was used to slowly inject 1 μL containing 200 ng of *PRPF31*-GFP and 36 µg of N-MSiNPs resuspended in PBS into the subretinal space. Finally, a drop of antibiotic (0.3% ciprofloxacin) was placed on each eye, and animals were kept on a 37 °C pad until they recovered from the anesthesia. Both eyes were injected. Mice were sacrificed by cervical dislocation one month after subretinal injections.

### 2.8. Funduscopic Examination

The mouse retinas were visualized in vivo three months after the subretinal injection using a retinal-imaging microscope (MICRON III, Phoenix Research Laboratories, Bend, OR, USA) as previously described [42]. A long-wavelength emission filter (transmission band Tavg N 93% 504.7–900 nm) and a short-wavelength excitation filter (486.5 nm transmission band Tavg N 90% 451.5) were used to detect the fluorescence signal of GFP.

### 2.9. OCT

After pupil dilation and sedation of the mice, retinal scans were taken using a Stratus time domain-optical coherence tomograph (Carl Zeiss, Jena, Germany). The acquisition protocol was a series of six equally spaced linear sweeps with a common central axis. The cuts were adjusted to explore 3 mm of the central retina, and the focus was adjusted to +12 diopters. The protocol for quantitative analysis included the retinal thickness and the measurement of the distance between the internal limiting membrane adjacent to the ganglion cell layer and the band joining the outer segments of photoreceptors with the RPE (OCT Stratus Software, Carl Zeiss, Germany). A retinal thickness map was used to represent the retinal thickness using a colorimetric scale.

### 2.10. Optomotor Test

Visual acuity was evaluated using the OptoMetry system (Cerebral Mechanics, Lethbridg, AB, Canada). To evaluate the visual acuity, 6 different spatial frequencies were used as follows: 0.031, 0.061, 0.092, 0.103, 0.194, and 0.272 cycles/degree (cyc/deg) at 100% of contrast sensitivity. For each frequency, a set of 20 random tests (10 clockwise and 10 counterclockwise) were performed per animal. When the stripes of the cylinder started rotating (12 degrees/s), the mouse moved the head in the same direction of stripe rotation (optokinetic reflex). The head movements of each animal were visualized through a video camera coupled to the software of the equipment. A red star cursor in the video frame indicated the head of the animal. This cursor allowed us to visualize the movements and served to fix the center of the virtual cylinder at a constant distance. The number of optokinetic reflexes elicited in the mice was registered.

### 2.11. ERG Recordings

ERG is used to measure the electrical response of retinal cells to light stimuli. Whole-field ERG was recorded in a Ganzfeld Color Dome (Diagnosys LCC, Lowell, MA, USA). To assess scotopic vision, mice were dark-adapted overnight. Anesthesia and pupil dilation of mice was performed as previously described [7]. A ring electrode made of gold wire (active electrode) was placed on the surface of the cornea, which was previously treated with a wetting agent (1% methylcellulose). Needle electrodes made of stainless steel were used as reference (forehead) and ground electrodes (tail). The narrowband filter was adjusted to frequencies of 0.312 to 300 Hz. A single flash white (6500 K) was used as a stimulus divided into 6 stages of increasing intensity at 0.01, 0.05, 0.2, 1, 3, and 10 candela (cd)·s/m^2^. Fifteen responses were recorded at each stage, with an interval of 15 s between each stimulus. To evaluate photopic vision, mice were adapted to light for 10 min with a background illumination of 30 cd/m^2^. The intensity of the stimulus was 3, 5, 10, 15, and 20 cd·s/m^2^. The amplitude and frequency of a- and b-waves were evaluated.

### 2.12. Immunofluorescence Experiments

The animals were euthanized by cervical dislocation, and the eyes were excised quickly and fixed in ice-cold 4% paraformaldehyde (PFA) in PBS, overnight, at 4 °C. The fixed eyes were then cryoprotected in 30% sucrose in PBS and embedded in optimal cutting temperature compound for cryotome sections. Serial sections of 18 μm thickness were mounted in five parallel series and processed for immunofluorescence [7]. Immunofluorescence experiments were performed on eyecup sections obtained from wild-type mice. The retinal sections were incubated overnight at 4 °C with the primary antibodies anti-PRPF31 (1:100; TA302582, OriGene Technologies Inc., Rockville, MD, USA) and anti-GFP (1:200; Cell Signaling 2956). After incubation, samples were washed 3 times in PBS 0.2% Tween 20 (PBS-T) and incubated with appropriate AlexaFluor secondary antibodies (Molecular Probes, Eugene, OR, USA) at room temperature for 1 h. After 3 washes, sections were mounted with Vectashield mounting medium containing DAPI (Vector Laboratories). Sections of all analyzed cases were processed in parallel following an identical protocol without the incubation step with the primary antibody, to be used as controls for immunoreaction specificity. Immunofluorescence experiments were also performed in cells grown on glass coverslips. Cells were fixed in 4% PFA and then permeabilized and blocked with 2% donkey serum/PBS-T for 1 h at room temperature. Incubation with primary antibodies anti-PRPF31 (1:100; OriGene Technologies Inc., TA302582) and anti-GFP (1:200; Cell signaling 2956) was performed for 1 h at room temperature. Cells were washed three times with PBS-T and incubated with AlexaFluor secondary antibodies (Molecular Probes). Coverslips were mounted on glass slides with Vectashield mounting medium containing DAPI (Vector Laboratories, Burlingame, CA, USA). Confocal images of retinal sections and cell coverslips were captured by a spectral confocal microscope TCS SP5 (Leica, Wetzlar, Germany) with an HCX PL APO Lambda blue 63 1.4 OIL objective, at 22 °C. Adobe Photoshop CS5.1 software was used for the digital amplification of the images.

### 2.13. Statistical Analysis

Experimental measurements were expressed as the means ± standard error of the mean and as median and interquartile range. The normal distribution of samples was evaluated by the Kolmogorov–Smirnov test. Statistically significant differences between groups were estimated by two-way ANOVA. A *p*-value < 0.05 was considered statistically significant.

## 3. Results

### 3.1. Synthesis and Characterization of the Nanoparticles

#### 3.1.1. Physico-Chemical Characterization

**Figure 1:**
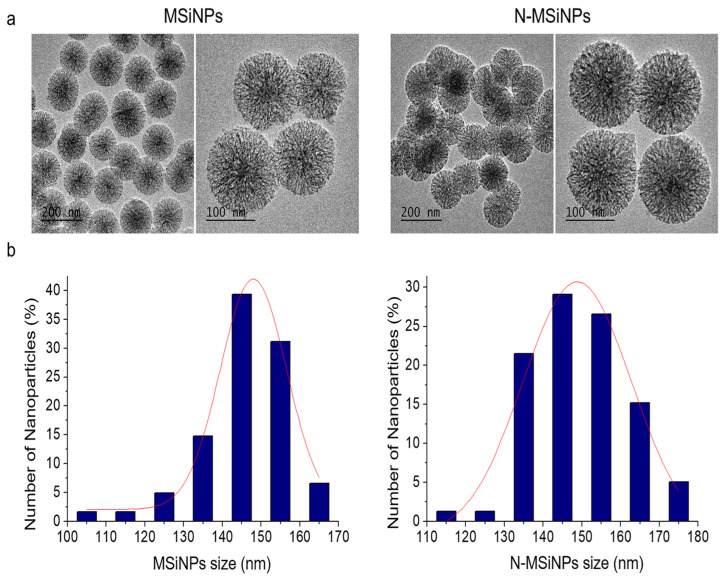
Nanoparticle morphology and particle size distribution; TEM images of the nanoparticles (**left**) and corresponding particle size distribution measured using micrograph image analysis (**right**): (**a**) MSiNPs and (**b**) N-MSiNPs.

MSiNPs were synthesized by the sol-gel method, as detailed in the Materials and Methods section. The X-ray fluorescence (XRF) quantitative analysis indicates 96.2 wt.% SiO_2_ and 3.8 wt.% CaO final binary composition. Transmission electron microscopy (TEM) comparative images of MSiNPs and N-MSiNPs are shown in Figure 1a,b, respectively. The two materials present homogeneous spherical morphology and size and display a well-resolved radial organized nanopore structure. Measured particle size distributions, also presented in Figure 1, show that functionalization brings about a particle size increase effect, showing a slight distribution broadening towards higher size values, although the medians at 146 nm and 149 nm with an interquartile range of 13 and 18 nm for MSiNPs and N-MSiNPs, respectively, are very close. In addition the TEM analysis revealed that amine functionalization does not affect final particle morphology and nanostructure.

**Figure 2:**
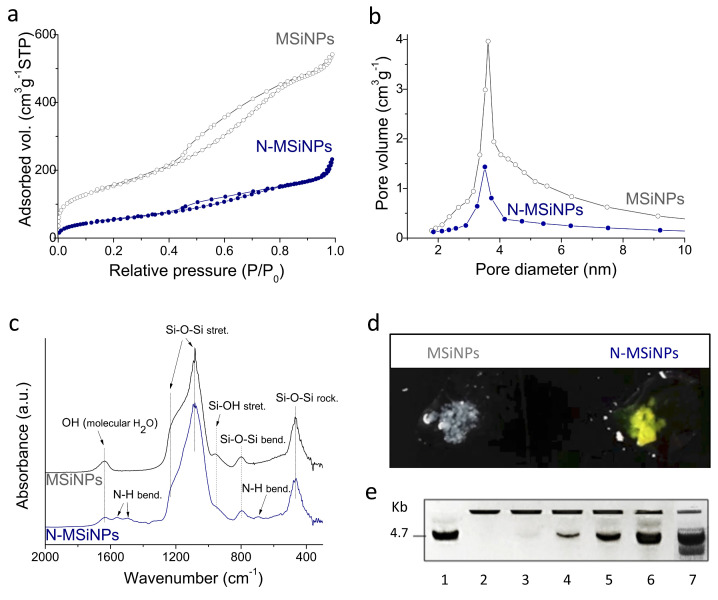
Physico-chemical characterization of the studied nanoparticle materials. (**a**) N_2_ adsorption–desorption isotherms. (**b**) Adsorption branch BJH pore size distributions. (**c**) FT-IR analysis. (**d**) Salicylaldehyde reaction analysis. (**e**) Electrophoresis gel image showing the retardation effect of essayed DNA plasmid, pEGFP-N1, after functionalized nanoparticles, N-MSiNPs, complexation using different pEGFP-N1: N-MSiNP (µg:µg) ratios: (1) 1:0 (DNA control sample), (2) 1:300, (3) 1:150, (4) 1:75, (5) 1:37.5, and (6) 1:18; and a second control sample using the non-functionalized nanoparticles, MSiNPs, (7) 1:300 pEGFP-N1:MSiNP ratio.

In contrast, N_2_ adsorption–desorption measurements indicate that functionalization produces an important outcome. Surface areas (S_BET_) of 476 ± 26 m^2^ g^−1^ and 176 ± 23 m^2^ g^−1^ and total pore volumes of 0.74 ± 0.05 cm^3^ g^−1^ and 0.28 ± 0.02 cm^3^ g^−1^ were measured, respectively, for MSiNP and N-MSiNP. Both materials exhibited type IV isotherms with type H1 hysteresis characteristics of accessible cylindrical channels with narrow size distribution. Likewise, the Barret-Joyner-Halenda (BJH) distribution for nanopore size shows in both cases the same maximum at 3.5 nm (Figure 2b). Therefore, NH_2_ functionalization significantly reduces the surface area and the pore volume parameters while preserving particle size and spherical morphology as well as nanopore size diameter.

The Fourier transform infrared (FT-IR) analysis of the nanoparticles (Figure 2c) shows a typical Si-O-Si bond vibrational mode characteristic of silica, SiO_2_. The main band at 1085 cm^−1^ corresponds to the Si-O-Si asymmetric stretching vibration, while the bands at 800 cm^−1^ and 467 cm^−1^ are ascribed respectively to the Si-O-Si bending and the rocking vibrations [43,44]. It is relevant to note that no signal detection for ethyl acetate (EA)-hexadecyltrimethyl ammonium bromide (CTAB) or nitrate groups confirms proper removal of the synthesis precursor reactants from the final nanoparticle product. As expected, N-MSiNPs show additional N-H bending mode vibration bands of the amino group at 1560 cm^−1^, 1492 cm^−1^, and 694 cm^−1^ [45]. In addition, the intensity peak at 955 cm^−1^, characteristic of Si-OH stretching vibration, is largely reduced for N-MSiNPs, indicating the reaction of Si-OH groups with 3-aminopropyl triethoxysilane (APTES) to produce the Si-O-Si linkage. Successful amino-functionalization of the nanoparticles was also confirmed by the salicylaldehyde test, showing N-MSiNPs turned yellow distinctively after a reaction of a few minutes, in comparison to the non-functionalized MSiNPs, which remained white (Figure 2d).

#### 3.1.2. DNA Loading Capacity

DNA loading capacity of the nanoparticles was evaluated using a gel retardation assay (Figure 2e) after incubation of N-MSiNPs and MSiNPs with the pEGFP-N1 plasmid, coding for the GFP fluorescent protein. DNA and nanoparticles were mixed at different ratios to determine the nanoparticles’ complexing capacity for DNA. The first lane was a control in which only DNA without nanoparticles was loaded, showing the migration of the free 4.7 Kb DNA molecule. In lane 2, where the plasmid:NP mixing ratio was 1:300, no free DNA band was observed; therefore, all DNA molecules were complexed with the N-MSiNPs and retained within the gel well. For the 1:150 ratio, a faint DNA band could be observed. In the next lanes, with increasing DNA rates, the nanoparticles’ loading capacity was overcome, and incremental amounts of free DNA were observed. Lane number 7 was a 1:300 ratio mixture of pEGFP but using non-functionalized MSiNPs, which resulted in the absence of complexation and therefore free DNA band mobility comparable to the DNA control of lane 1. The experiments proved that amino-functionalization allowed an effective complexation of the plasmid DNA using N-MSiNPs when the nanoparticle amount was sufficient. The pEGFP:N-MSiNP ratio of 1:180 was considered suitable and then used further for the subsequent biological experimentation. The experiments also confirmed that the amino-functionalization, whilst reversing negative intrinsic silica surface charge to positive, is a key synthesis step for mesoporous silica-based nanoparticles to load negatively charged DNA [45].

### 3.2. In Vivo Studies on the Safety of Subretinal Administration

#### 3.2.1. Structural Preservation of the Retina

As the main purpose for the design of N-MSiNPs was its future use as a vehicle to transfect genetic material into mammalian cells, an initial assessment of the safety of N-MSiNPs for in vivo use was performed on an animal model. Subretinal administration of nanoparticles loaded with plasmid DNA (pEGFP/N-MSiNPs, named GFP/N-MSiNPs) was evaluated in adult mice. Specifically, 3- to 4-month-old c57bl/6 mice received, in both eyes, subretinal injection of 1 µL of a suspension containing the GFP/N-MSiNPs complex in saline phosphate buffer (PBS), or an equal amount of empty N-MSiNPs, or PBS alone. A complete ophthalmic evaluation was performed three months after the intervention.

**Figure 3:**
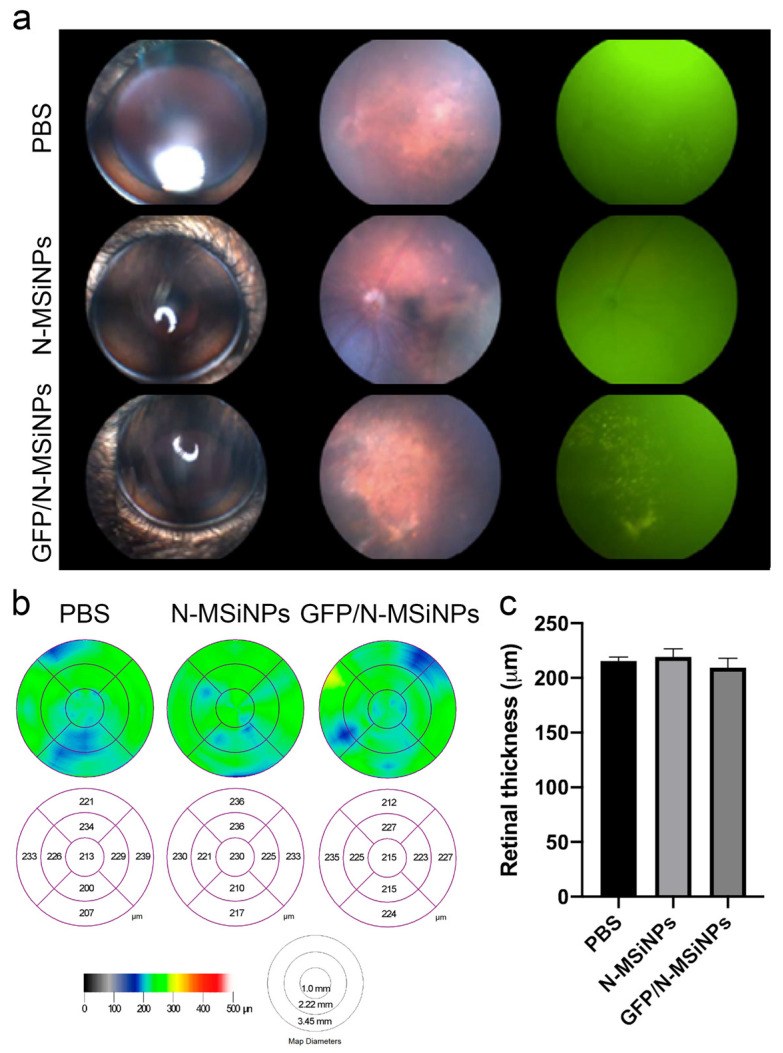
Morphological evaluation of mouse eyes three months after subretinal injection of 1 µL PBS, empty nanoparticles (N-MSiNPs), or nanoparticles loaded with the pEGFP-N1 plasmid (GFP/N-MSiNPs). (**a**) External aspect of the cornea (left column), eye fundus in the visible spectrum (central column), and fluorescent spectrum (right column). (**b**) OCT images showing the retinal thickness are represented as a colorimetric scale in the retinal maps. (**c**) Quantification of the retinal thickness measured by OCT. Bars in (**c**) represent retinal thickness in µm ± standard error of the mean (SEM).

The health of the retinal tissue and any possible degenerative signs were assessed by studying fundus images. Fundus fluorescence images allow for the detection of the local expression of the fluorescent GFP protein. Figure 3a shows the safety of N-MSiNPs applied to the subretinal space. The fundus images display no differences in retinal integrity after the injection of N-MSiNPs, empty or loaded with DNA, when compared with the PBS-injected eyes. In Figure 3a, images of the external aspect of injected eyes are in the first column, the fundus images in the visible spectrum are displayed in the second column, and the fundus images for fluorescence visualization in the third column. GFP expression detected as fluorescent dots in the third column was apparent in the eyes injected with GFP/N-MSiNPs, indicating successful delivery of the genetic material into the cells of the retina and the functional expression of the GFP transgene. In all the cases, the injection of nanoparticles did not adversely affect the integrity of the retinal tissue.

Another ophthalmological test that reflects the health of retinal tissue is the measurement, by optical coherence tomography (OCT), of the thickness of the cell layers that compose the tissue. In Figure 3b,c, some representative OCTs are shown along with the quantification of the OCT measurements, indicating that this parameter is also unaffected by the injection of the nanoparticles.

**Figure 4:**
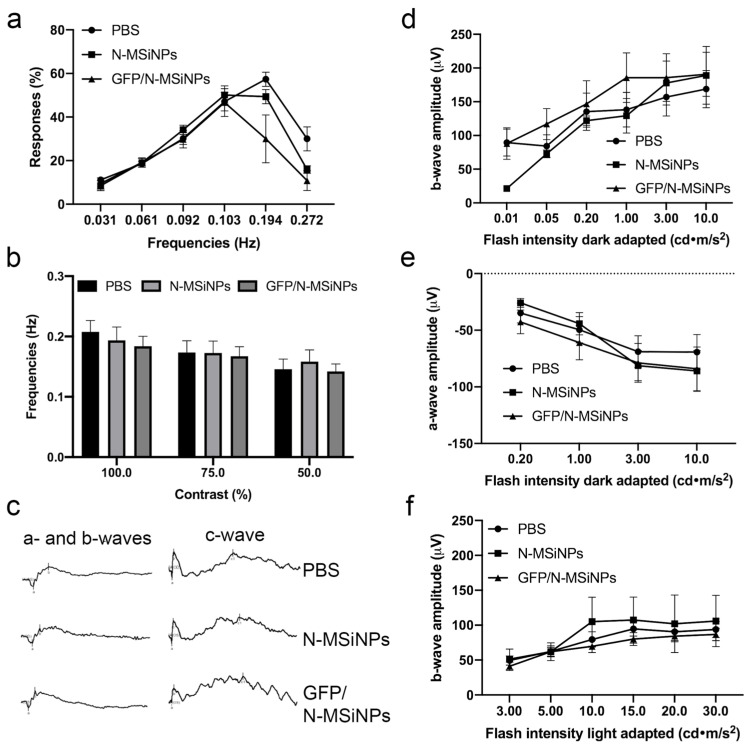
Functional evaluation of mouse retinas three months after subretinal injection of 1 µL PBS, empty nanoparticles (N-MSiNPs), or nanoparticles loaded with the pEGFP-N1 plasmid (GFP/N-MSiNPs). (**a**) Visual acuity measured by optomotor test at 100% contrast sensitivity. Values in (**a**) represent percentages of optokinetic responses ± SEM at different spatial frequencies (*n* = 6). (**b**) Contrast perception threshold measured at 100%, 75%, and 50% contrast sensitivity. Bars in (**b**) represent the highest spatial frequencies in which optokinetic responses were visualized ± SEM. (**c**) Representative ERG traces observed in injected mice. The electrical activity of photoreceptors (a-and b-waves) and RPE (c-wave) are shown. (**d**,**e**) Quantification of the amplitude of the ERG b-wave and a-wave in scotopic conditions (dark-adapted), reflecting the rod and cone/rod activity, respectively. (**f**) Quantification of the amplitude of the ERG b-wave in photopic conditions (light-adapted), reflecting the activity of cone photoreceptors. Values in (**d**–**f**) represent wave amplitude in µV ± SEM at different flash intensities.

The visual function of the injected animals was evaluated using the optomotor test, measuring visual acuity. In this test, the optokinetic reflex was measured using the responses to visual stimuli in the form of movements of the animal’s head to follow the rotary motion of a pattern of contrasting lines (light and dark stripes). The percentage of responses relates to the visual acuity of the animals. The contrast of the band pattern is modulable, allowing the measurement of the contrast perception threshold. Figure 4a shows the visual acuity at 100% contrast presenting no difference between the different treatments. Visual acuity was not affected by the treatment in the frequency range of the test. Finally, the effect of the injections on the threshold of frequencies that the animals can perceive was also determined, carrying out the optokinetic test at different contrasts of 100%, 75%, and 50%. Figure 4b shows that the injection of N-MSiNPs, with or without genetic load, did not significantly affect the perception threshold for the animals tested.

#### 3.2.2. Functional Study of the Retina by ERG

Additional in vivo evaluation of the physiological activity of the treated eyes was performed by electroretinogram (ERG) to detect the electrical activity of the retinal cells in response to light stimuli. Figure 4c shows representative a-, b-, and c-waves of the ERG traces in animals treated with PBS vehicle (upper traces), empty nanoparticles (intermediate traces), or nanoparticles loaded with the genetic material (bottom traces). In none of the treated groups, the ERG waves showed significant differences. The amplitude of these waves was quantified at different light intensities and analyzed in more detail in Figure 4d–f. Figure 4d shows the amplitudes of the b-wave generated by the rod photoreceptor cells in response to different light intensities in dark-adapted animals. Figure 4e displays the amplitudes of a-wave representing the combined response of cone and rod cells in dark-adapted animals. Figure 4f represents the amplitude of b-wave for light-adapted animals, which measures the response of the cones alone. According to the data, there was no difference when comparing the three conditions. In conclusion, the subretinal injection of nanoparticles did not produce any negative effect in terms of the physiological photoreceptor electrical activity.

In summary, in vivo application of the studied nanoparticles as a potential non-viral vector for retinal delivery showed safety after a complete panel of ophthalmic tests. The GFP/N-MSiNP–treated eyes showed GFP expression in the fundus along with preservation of retinal morphology, with conserved retinal thickness and structure compared to eyes treated with unloaded particles or PBS injection. In the tested conditions, nanoparticles did not induce any degenerative process in the mouse retina. Additionally, functional studies on the photoreceptor responses to light stimuli and the visual acuity of the treated animals revealed that the treatment had no detrimental effect on the sensory function of the photoreceptors nor the contrast perception nor visual acuity of the animals. Therefore, these nanoparticles demonstrated biocompatibility and safety for delivering genes to the subretinal space in a preclinical setting.

### 3.3. In Vitro Studies in Human Cells

To assess the cytocompatibility and the ability of N-MSiNPs to deliver genetic material into a living human cell, the HEK-293 cell line was used for in vitro transfection using these nanoparticles. N-MSiNPs were loaded with plasmid DNA prepared to express the human *PRPF31* gene, which is a candidate gene for RP gene therapy. This gene encodes a splicing factor, expressed predominantly in the cell nucleus. In plasmid design, the *PRPF31* transgene was fused to GFP (*PRPF31*-GFP) for easy detection of fluorescence expression. The transfection was performed with an equal amount of *PRPF31*-GFP construct either complexed to the N-MSiNPs or delivered into the cells using Lipofectamine as the standard transfection method. Results were analyzed by immunofluorescence and Western blot (WB).

**Figure 5:**
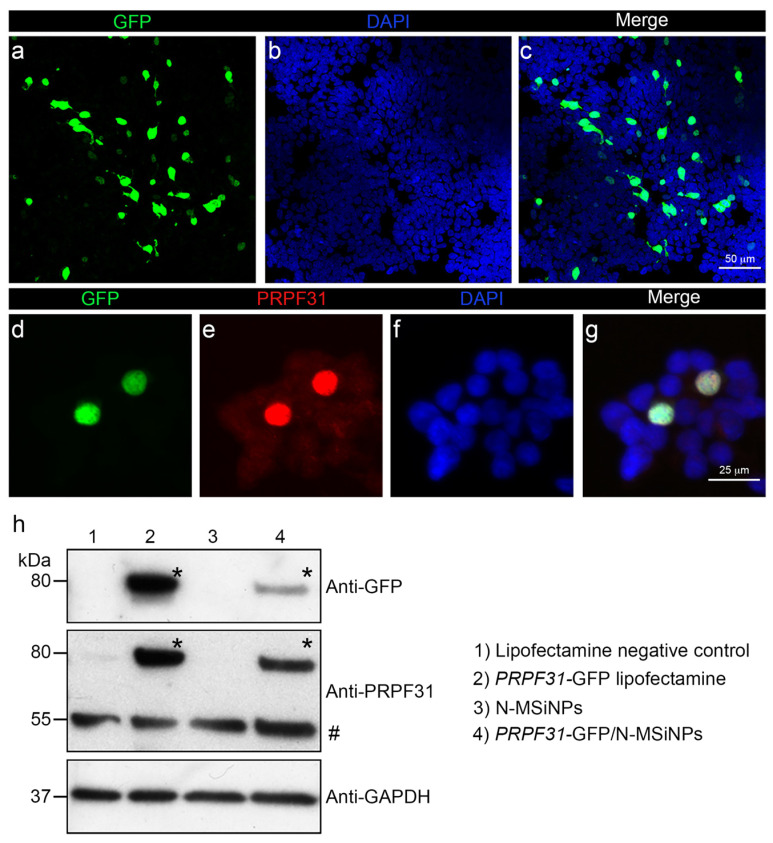
Transfection efficacy of *PRPF31* gene using the nanoparticle delivery system *PRPF31*-GFP/N-MSiNPs in a human cell line. Panels (**a**–**g**), immunofluorescence images of HEK-293 cells transfected with N-MSiNPs loaded with the *PRPF31*-GFP plasmid. GFP fluorescent signal (**a**,**d**), in green; anti-PRPF31 fluorescent signal (**e**), in red. Cell nuclei were stained with DAPI (**b**,**f**), in blue. Merged images of the different channels are shown in (**c**,**g**). (**h**) WB of HEK-293 transfected cells showing the expression levels of the *PRPF31* transgene fused to GFP. Protein extracts of HEK-293 treated with only Lipofectamine (negative control), in lane 1; from cells transfected with *PRPF31*-GFP using Lipofectamine, in lane 2; from cells incubated with empty N-MSiNPs, in lane 3; and from cells transfected with N-MSiNPs loaded with *PRPF31*-GFP, in lane 4. WBs were performed with anti-GFP (upper panel) and anti-PRPF31 (central panel) antibodies. GAPDH was used as a loading control (bottom panel). The (*) in the upper and central panels marks the expected molecular weight (80 kDa) of the PRPF31-GFP fused protein visualized by anti-GFP and ant-PRPF31 antibodies. The (#) in the central panel marks the expected molecular weight (55 kDa) of the endogenous PRPF31 protein. Scale bars represent 25 and 50 µm.

Figure 5 shows that N-MSiNPs loaded with the plasmid were able to transfect human cells in vitro (19% of transfection efficacy; Supplementary Figure S1), leading to the expression of the exogenous gene and the correct localization of the PRPF31-GFP protein in the cell nuclei. The immunofluorescence image in the upper panel showed a green GFP signal corresponding to the expression of the exogenous *PRPF31* gene fused to GFP in HEK-293 cells (Figure 5a,c). Detailed images (Figure 5d,e) depict the co-localization of the GFP green signal with the red immunofluorescence corresponding to *PRPF31* (Figure 5e) in the cell nuclei. These results were confirmed by WB (Figure 5h). An 80 kDa band corresponding to the molecular weight of the *PRPF31*-GFP fused protein was present in the anti-*PRPF31* and anti-GFP WBs. Expression driven by exogenous genetic material was only observed in the extracts of cells transfected with Lipofectamine or in those transfected with N-MSiNPs loaded with plasmid. A higher level of expression of the exogenous gene was observed for Lipofectamine-transfected cells. Endogenous PRPF31 protein was detected as a 55 kDa immunoreactive band. The nanoparticles have proven biocompatibility with human cells and the ability to deliver a potentially therapeutic transgene.

### 3.4. In Vivo Administration of a Therapeutic Gene

**Figure 6:**
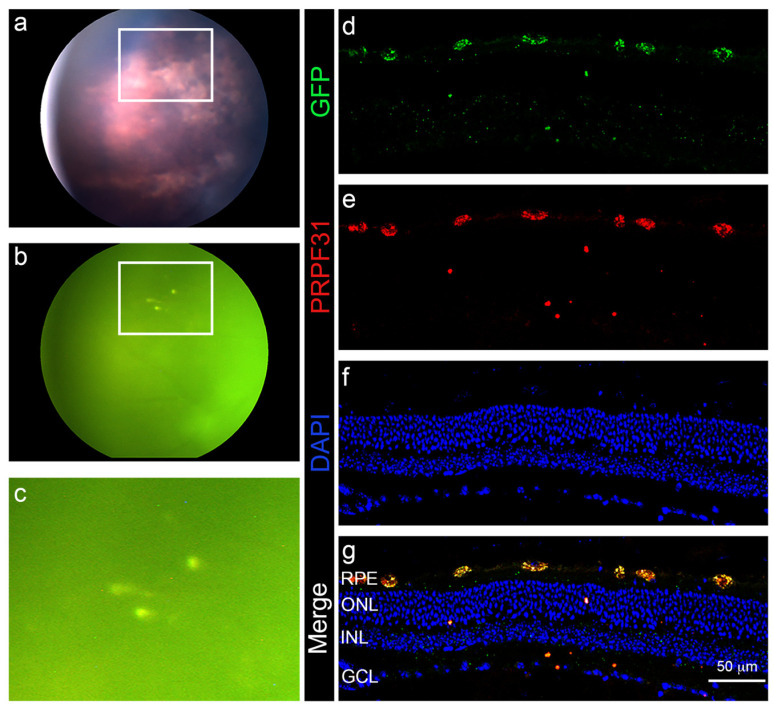
Fundus images and the retinal section of *PRPF31*-GFP/N-MSiNP subretinally injected mice. (**a**) Fundus image. (**b**) Fluorescence fundus. (**c**) Detail of the injection site showing GFP fluorescent dots. Immunostaining of retinal sections for GFP (**d**), in green; PRPF31 (**e**), in red. Nuclei stained with DAPI (**f**), in blue. (**g**) Merged images of the different channels. GFP and PRPF31 signal co-localized mainly in RPE cells. RPE = retinal pigment epithelium, ONL = outer nuclear layer, INL = inner nuclear layer, GCL = ganglion cell layer. Scale bar represents 50 µm.

To evaluate the ability of N-MSiNPs to deliver a candidate therapeutic transgene into the retina of a living animal model, *PRPF31*-GFP loaded N-MSiNPs were used to transfect retinal cells in 2-month-old c57bl/6 wild-type mice. The mice were subretinally injected with a 1µL of a suspension of *PRPF31*-GFP/N-MSiNPs in PBS. One month after the intervention, the treated eyes were examined by funduscopy (Figure 6a–c), finding no signs of abnormal retinal morphology or damage. Fundus fluorescence was also examined to evaluate fluorescent protein expression in the living tissue, finding GFP fluorescence at the injection site. Immediately after the ophthalmic evaluation, mice were sacrificed to obtain retinal tissue sections and examine the presence of transgene expression driven by the N-MSiNP delivery. Immunofluorescence for GFP (Figure 6d) and PRPF31 (Figure 6e) regarding the retinal layers marked with nuclear staining (Figure 6f,g) showed the co-localization of both signals in the retinal pigmented epithelium (RPE) layer of the retina (Figure 6e), demonstrating that the transgene was effectively delivered to and expressed in this retinal cell layer. A detailed version of Figure 5 showing the expression of both the transgene and endogenous PRPF31 is found in Supplementary Figure S2. The transfection did not affect the distribution or localization of the tight junctions in the RPE (Supplementary Figure S3). From these tests, it can be assumed that these nanoparticles can deliver a gene of therapeutic potential to the RPE cells of experimental animals.

## 4. Discussion

Reproducible MSiNPs with a final composition of 96.2% SiO_2_/3.8% CaO in weight were obtained. The functionalization of the nanoparticles with NH_2_ groups using APTES was successfully carried out as the salicylaldehyde reaction shows. The MSiNPs were white, and N-MSiNPs turned yellow due to the reaction of amino groups with the salicylaldehyde. In addition, FT-IR confirmed the presence of amino groups for N-MSiNPs, which cannot be observed in the MSiNP spectrum. The nanoparticles presented a spherical morphology with radial nanopores for both cases, MSiNPs and N-MSiNPs. MSiNP size did not vary after functionalization, as TEM images and the size graphic showed, keeping dimensions of approximately 135 nm. The high specific surface and pore volume of the MSiNPs were affected by functionalization, since both of them change (from 483 ± 51 m^2^ g^−1^ and 0.74 ± 0.05 cm^3^ g^−1^ for MSiNPs to 176 ± 23 m^2^ g^−1^ and 0.28 ± 0.02 cm^3^ g^−1^ for N-MSiNPs). There was no significant change in the pore size, being 3.5 nm for both nanoparticles. The MSiNPs hardly presented DNA loading, whereas 1 ng of pEGFP-N1 plasmid was successfully loaded by 180 ng of N-MSiNPs. Therefore, amino-functionalization played an important role in the properties of the nanoparticles, as systems for loading and releasing DNA reversing their charge from negative to positive and promoting the loading of the negatively charged DNA by the positive charged N-MSiNPs [45].

Once the ability of the N-MSiNPs to load DNA was established, a thorough evaluation of the safety of this material for retinal delivery was performed. A complete panel of ophthalmic tests was used to determine the safety of the delivery of a mammalian expression vector into the subretinal space of mice using N-MSiNPS as a non-viral vector. The preservation of retinal morphology, thickness, and structure was demonstrated by funduscopy imaging and OCT tests, showing no significant differences among GFP/N-MSiNPs, empty N-MSiNPs, or PBS-treated animals. The GFP/N-MSiNPs treated eyes showed GFP expression in the fundus. In the tested conditions, the injection of these nanoparticles did not induce any degenerative process in the mouse eyes. Additionally, functional studies were performed to assess the effects of the intervention on the photoreceptor responses to light stimuli and the visual acuity of the treated animals, revealing that the treatment had no detrimental effect on the sensory function of the photoreceptors nor on the contrast perception or visual acuity of the animals. Therefore, these nanoparticles proved to be safe for the delivery of transgenes to the subretinal space in a preclinical setting. In summary, the injection of nanoparticles with or without genetic load had no detrimental effect on the visual function of the examined animals.

The N-MSiNPs also showed biocompatibility and capacity to transfect human cells in vitro when HEK-293 cells were treated with *PRPF31*-GFP/N-MSiNPs. Immunostaining and WB indicated the expression of the exogenous PRPF31 protein fused to GFP, which also localized in the expected cell compartment. To compare the expression of the PRPF31-GFP construct in the HEK-293 cells, we used the N-MSiNP complex or Lipofectamine 2000. The transgene expression was slightly higher with the standard transfection method using Lipofectamine. However, for these types of comparative studies, it would be advisable to use optimized reagents to transfect human cells such as Lipofectamine 3000 [46].

Finally, the functionalized nanoparticles demonstrated the transfection of RPE cells of mice in vivo. One month after subretinal injection with *PRPF31*-GFP loaded N-MSiNPs, the mice retinal sections revealed that the expression of the PRPF31-GFP protein localized in the RPE cell layer. Although the PRPF31 protein was ubiquitously expressed, it has been shown in several mammalian species, including humans, that the protein is highly expressed in the RPE compared to the neuroretina [7]. In addition, no abnormal morphology or damage was observed by funduscopy in the retina of the transfected mice. Thus, these nanoparticles can deliver a potentially therapeutic transgene to the RPE cells of experimental animals.

The presented results using N-MSiNPs indicate that these systems can deliver DNA in the form of a mammalian expression vector into the subretinal space of mice without any detrimental effect on the health of the retinal tissue. An example of delivery of a possible therapeutic transgene was shown, driving its expression to the RPE, which is the target cell type for many retinal diseases, including *PRPF31*-RP. For some genetic diseases, such as autosomal-dominant RP caused by mutations in *PRPF31*, the disease mechanism involves a dominant-negative effect plus haploinsufficiency, and the silencing of the mutated allele along with the required dose of the gene may be required to obtain a therapeutic effect. In this respect, non-viral delivery of genetic material offers a broader range of applications than AAVs. Functionalized-mesoporous silica nanoparticles have already shown the capacity for efficient dual-delivery of siRNA and plasmid DNA into mammalian cells without cytotoxicity [47], thus providing support for the possible use of these materials in dual-gene therapy. This work proves that N-MSiNPs are able to safely transfect RPE cells and thus might be useful for gene release in degenerative pathologies of the retina that primarily affect the RPE, such as Stargardt’s disease or age-related macular degeneration.

The biomedical applications of the MSiNPs have already been studied for bone regeneration. Lee et al. studied the effects of MSiNPs on the differentiation of dental pulp stem cells [45]. Inhibition of osteoclastogenesis through siRNA delivery with tuneable mesoporous bioactive nanocarriers was studied by Kim et al. [48]. In addition, due to properties such as sustained and local drug delivery, MSiNPs are promising platforms for cancer research [30]. Shoaib et al. [49] prepared mesoporous nano-bioglass for the release of imatinib and for studying the in vitro inhibitory effects on osteosarcoma cancer cells. Osteogenic silver oxide–doped mesoporous bioactive glass has been studied for the controlled release of doxorubicin against the bone cancer cell line (MG-63) [50]. In the case of cancer, mesoporous silica nanoparticles conjugated with folic acid have presented good results for enhancing the therapeutic efficacy of topotecan in retinal cancers [33].

Here, MSiNPs were successfully used as a safe delivery platform for genes into the retina in a preclinical setting. Although further work is needed to validate the therapeutic utility of MSiNPs for gene therapy, this first report of MSiNPs as a gene delivery platform for ocular applications opens a promising field for future applications.

## 5. Conclusions

Amino-functionalized mesoporous silica-based nanoparticles, N-MSiNPs, of homogeneous spherical shape, diameter sizes of around 150 nm, and radially oriented nanopores of 3.5 nm were synthesized and proved to have excellent capacity to load plasmid DNA. These nanoparticles were tested for gene delivery into the subretinal space of mice, showing no evidence of adverse effects regarding the integrity of the retinal tissue and the visual function. The exogenous *PRPF31*-GFP transgene was correctly delivered by N-MSiNPs to the nucleus of human cells in vitro and to the RPE cell layer of mouse retina after subretinal injection. Our results indicate that N-MSiNPs could represent a safe vehicle for the release of potentially therapeutic genes to the retina and may constitute an advanced alternative to viral vectors for gene therapy of inherited retinal degenerative diseases such as RP.

## 6. Patents

Patent number: P202031290. Date: 23 December 2020. 11:49 (CET). Reference: ES1641.1642. Country: ES. Title: Vectores no virales a partir de nanopartículas mesoporosas para su aplicación en terapia génica para el tratamiento de patologías degenerativas de la retina.

## Data Availability

Data are contained within the article.

## Supplementary Materials

The following supporting information can be downloaded at: https://www.mdpi.com/article/10.3390/jcm11082170/s1, Figure S1: Percentage of HEK-293 cells transfected with PRPF31-GFP/N-MSiNPs; Figure S2: Endogenous expression of prpf31 protein in a c57bl/6 wild type mouse retina; Figure S3: The transgene expression driven by N-MsiNPs delivery does not affect the localization and distribution of ZO1 in RPE cells in vivo.
