# Supplementary material for: Mesoporous Silica-Based Nanoparticles as Non-Viral Gene Delivery Platform for Treating Retinitis Pigmentosa
Source: J Clin Med. 2022 Apr 13;11(8):2170. doi: 10.3390/jcm11082170 (PMC9026300; doi:10.3390/jcm11082170)

Supplementary Figure S1. Percentage of HEK-293 cells transfected with PRPF31-GFP/N-MSiNPs. Cell nuclei were stained with DAPI to quantify the total number of cells in each replicate, and the number of GFP-positive cells was quantified. The graph shows the percentage of GFP-positive and GFP-negative cells in three replicates. The estimated mean of transfection efficacy was 19%. Cell nuclei counting was performed with ImageJ software.

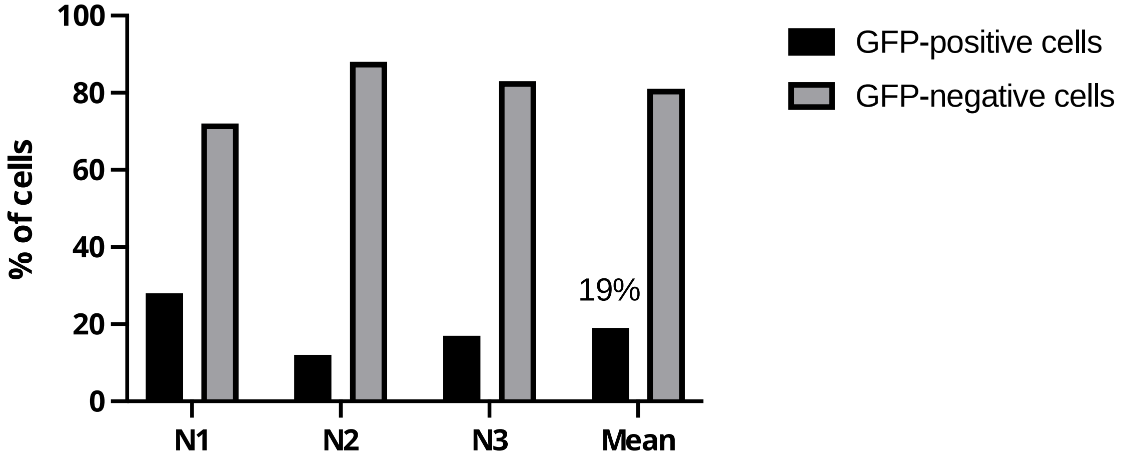

Supplement: Supplementary file 1 [file jcm-11-02170-s001.zip › Supplementary Figure S1.pdf]
