# Supplementary material for: Mesoporous Silica-Based Nanoparticles as Non-Viral Gene Delivery Platform for Treating Retinitis Pigmentosa
Source: J Clin Med. 2022 Apr 13;11(8):2170. doi: 10.3390/jcm11082170 (PMC9026300; doi:10.3390/jcm11082170)

Supplementary Figure S2. Endogenous expression of prpf31 protein in a c57bl/6 wild type mouse retina (a-b). Black and white magnified images (c-e) of the Figure 6 d-f, where the expression of the transfected PRPF31-GFP/N-MSiNPs (arrows) and endogenous prpf31 (arrowheads) can be observed in the retinal pigment epithelium (RPE). ONL= outer nuclear layer. Scale bars represent 50 µm.

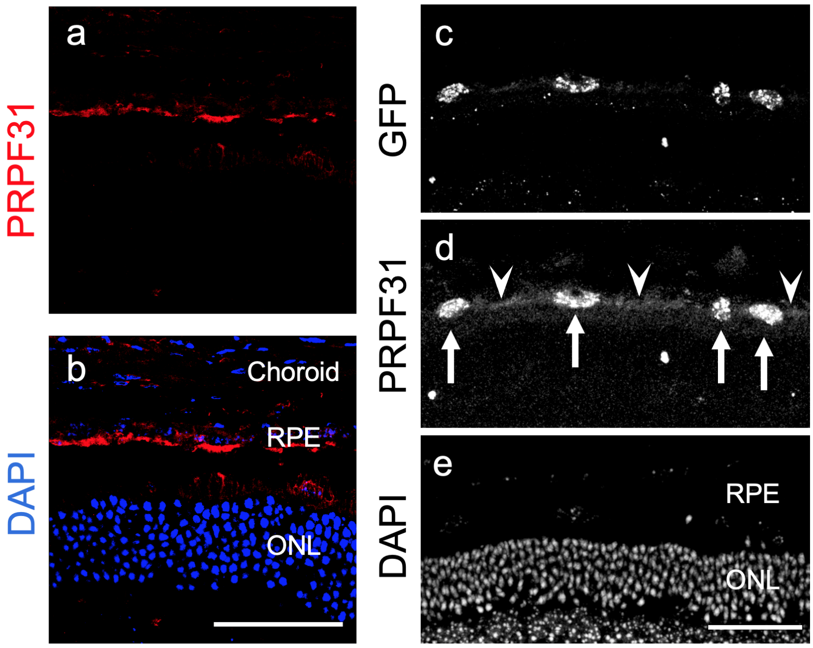

Supplement: Supplementary file 1 [file jcm-11-02170-s001.zip › Supplementary Figure S2.pdf]
