# Supplementary material for: Mesoporous Silica-Based Nanoparticles as Non-Viral Gene Delivery Platform for Treating Retinitis Pigmentosa
Source: J Clin Med. 2022 Apr 13;11(8):2170. doi: 10.3390/jcm11082170 (PMC9026300; doi:10.3390/jcm11082170)

Supplementary Figure S3: The transgene expression driven by N-MsiNPs delivery does not affect the localization and distribution of ZO1 in RPE cells *in vivo*. Expression of GFP protein (b, f) and ZO1 (c, g) in c57bl/6 wild-type mouse retina. In the upper row, images of a non-injected control eye. In the lower row, images of an eye injected with PRPF31-GFP/N-MsiNPs. Nuclei stained with DAPI (a, e) allow visualization of the retinal structure. INL= inner nuclear layer; ONL= outer nuclear layer; RPE= Retinal pigment epithelium. Scale bars represent 50 µm.

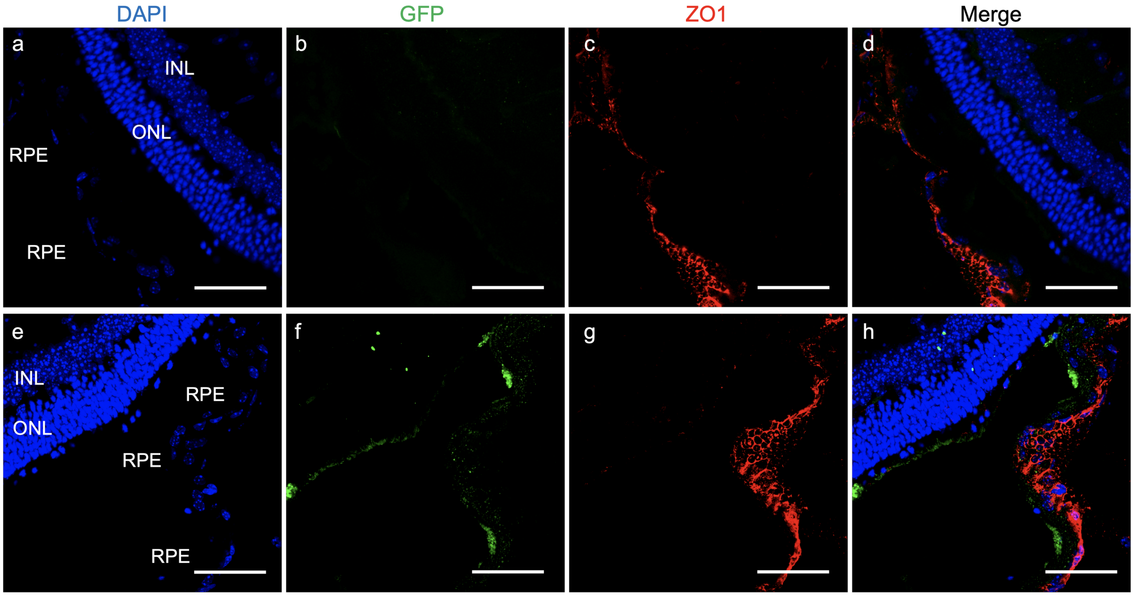

Supplement: Supplementary file 1 [file jcm-11-02170-s001.zip › Supplementary Figure S3.pdf]
